# Establishment of markerless gene deletion tools in thermophilic *Bacillus smithii* and construction of multiple mutant strains

**DOI:** 10.1186/s12934-015-0286-5

**Published:** 2015-07-07

**Authors:** Elleke F Bosma, Antonius H P van de Weijer, Laurens van der Vlist, Willem M de Vos, John van der Oost, Richard van Kranenburg

**Affiliations:** Laboratory of Microbiology, Wageningen University, Dreijenplein 10, 6703 HB Wageningen, The Netherlands; Corbion, Arkelsedijk 46, 4206 AC Gorinchem, The Netherlands

**Keywords:** *Bacillus smithii*, Thermophile, Lactate dehydrogenase, Sporulation, Pyruvate dehydrogenase, Counter-selection system

## Abstract

**Background:**

Microbial conversion of biomass to fuels or chemicals is an attractive alternative for fossil-based fuels and chemicals. Thermophilic microorganisms have several operational advantages as a production host over mesophilic organisms, such as low cooling costs, reduced contamination risks and a process temperature matching that of commercial hydrolytic enzymes, enabling simultaneous saccharification and fermentation at higher efficiencies and with less enzymes. However, genetic tools for biotechnologically relevant thermophiles are still in their infancy. In this study we developed a markerless gene deletion method for the thermophile *Bacillus smithii* and we report the first metabolic engineering of this species as a potential platform organism.

**Results:**

Clean deletions of the *ldhL* gene were made in two *B. smithii* strains (DSM 4216^T^ and compost isolate ET 138) by homologous recombination. Whereas both wild-type strains produced mainly l-lactate, deletion of the *ldhL* gene blocked l-lactate production and caused impaired anaerobic growth and acid production. To facilitate the mutagenesis process, we established a counter-selection system for efficient plasmid removal based on *lacZ*-mediated X-gal toxicity. This counter-selection system was applied to construct a sporulation-deficient *B. smithii* Δ*ldhL* Δ*sigF* mutant strain. Next, we demonstrated that the system can be used repetitively by creating *B. smithii* triple mutant strain ET 138 Δ*ldhL* Δ*sigF* Δ*pdhA*, from which also the gene encoding the α-subunit of the E1 component of the pyruvate dehydrogenase complex is deleted. This triple mutant strain produced no acetate and is auxotrophic for acetate, indicating that pyruvate dehydrogenase is the major route from pyruvate to acetyl-CoA.

**Conclusions:**

In this study, we developed a markerless gene deletion method including a counter-selection system for thermophilic *B. smithii*, constituting the first report of metabolic engineering in this species. The described markerless gene deletion system paves the way for more extensive metabolic engineering of *B. smithii*. This enables the development of this species into a platform organism and provides tools for studying its metabolism, which appears to be different from its close relatives such as *B. coagulans* and other bacilli.

**Electronic supplementary material:**

The online version of this article (doi:10.1186/s12934-015-0286-5) contains supplementary material, which is available to authorized users.

## Background

Microbial conversion of biomass to fuels such as ethanol or hydrogen, or to green chemical building blocks such as organic acids has gained increasing attention over the last decade [1, 2]. Thermophilic microorganisms have several advantages over mesophilic organisms for use as microbial production hosts. Fermentation at high temperatures lowers cooling costs and contamination risks and increases product and substrate solubility [3–5]. Moreover, the optimum temperature of moderate thermophiles matches that of commercial hydrolytic enzymes, enabling simultaneous saccharification and fermentation at higher efficiencies and with less enzymes compared to mesophilic bacteria [6].

Despite the aforementioned advantages of thermophiles, mesophilic model organisms such as *Escherichia coli* and *Saccharomyces cerevisiae* are still preferred production organisms, as these are well-studied and genetic tools are available to enable their use as versatile platform organisms [7, 8]. Genetic tools for biotechnologically relevant thermophiles are recently emerging for different species, but most are still in their infancy or highly strain-specific. Several strictly and facultatively anaerobic thermophiles have been engineered for green chemical and fuel production, as has been reviewed recently [9, 10]. Most engineering efforts in thermophiles have so far been directed at ethanol production, but recently also examples for chemical production have been shown such as *Thermoanaerobacterium aotearoense* for lactate production [11], *Bacillus licheniformis* for 2,3-butanediol production [12], and *Bacillus coagulans* for d-lactate production [13, 14]. The development of genetic tools for thermophilic organisms is crucial to fully understand their metabolic versatility and to establish a thermophilic production platform for green chemical and fuel production. For industrial applications, markerless gene deletions should be made such that no antibiotic resistance genes or other scars are introduced into the target genome. This is especially important when working with thermophilic organisms as the number of available markers is limited, requiring re-use of the marker [9, 10].

Recently, we isolated a thermophilic *Bacillus smithii* strain capable of degrading C5 and C6 sugars at a wide range of temperatures and pHs [15] and demonstrated electrotransformation of several *B. smithii* strains with plasmid pNW33n. In the current study, we developed a clean gene deletion method and counter-selection system for this species and applied this to create multiple markerless gene deletions both in the previously isolated *B. smithii* ET 138 [15] and in the type strain *B. smithii* DSM 4216^T^.

## Results

### Construction of markerless *ldhL* deletion mutants

**Figure 1:**
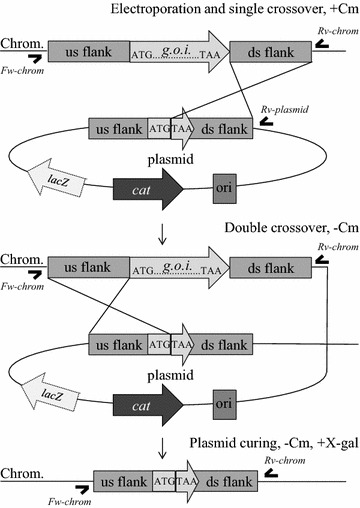
Illustration of markerless knockout construction in *B. smithii*. **a** pNW33n-derived plasmid containing the 1,000 bp-flanking regions of a gene of interest (‘*g.o.i.*’) is introduced into the cell via electroporation, plated on LB2 containing chloramphenicol at 55°C and subjected to PCR analysis to check for single crossovers using primers ‘Fw-chrom’ and ‘Rv-plasmid’. Colonies containing single crossovers are transferred to plates without chloramphenicol, after which PCR screening is performed to check for double crossovers using primers ‘Fw-chrom’ and ‘Rv-chrom’. If the 2nd recombination occurs via the same flank as the 1st recombination, a wild-type genotype will be the result. If the 2nd recombination occurs via the other flank, this results in a knockout genotype. To delete the *ldhL* gene, only PCR screening was performed and no *lacZ* gene was present on the plasmid. For creating the *sigF* and *pdhA* mutants, the plasmid also contained the *lacZ* gene. In those cases, *lacZ* counter-selection was performed by plating an overnight culture on LB2 plates containing 100 µg/mL X-gal, resulting in toxic concentrations of the X-gal cleavage product in the presence of *lacZ,* resulting in small blue colonies still containing the plasmid (either replicating plasmid or inserted in the genome via single crossover) and white colonies that have cured the plasmid. *g.o.i.* gene of interest, *cat* chloramphenicol acetyltransferase, *chrom.* chromosome, *us* upstream, *ds* downstream, *Cm* chloramphenicol, primers are indicated with *black hooks.*

**Figure 2:**
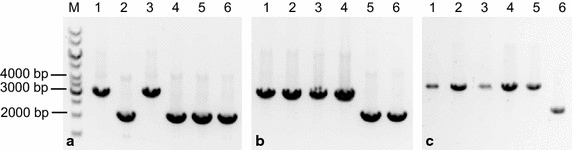
Gel-electrophoresis of PCR products from amplified target genes *ldhL* (**a**), *sigF* (**b**) and *pdhA* (**c**). Order of strains in all three parts of the picture: M: Thermo Scientific 1 kb DNA ladder, *1* DSM 4216^T^ wild-type, *2* DSM 4216^T^ Δ*ldhL*, *3* ET 138 wild-type, *4* ET 138 Δ*ldhL*, *5* ET 138 Δ*ldhL* Δ*sigF*, *6* ET 138 Δ*ldhL* Δ*sigF* Δ*pdhA*. The original gel pictures without cropping are provided in Additional files 1 (for **a**, **b**) and 2 (for **c**). **a** Amplification of the region 1,000 bp up- and downstream of the *ldhL* gene using primers BG 3663 and 3669. The wild-type genotype results in a product of 3,036 bp, whereas the complete deletion of the *ldhL* gene is confirmed by a shift of the product to 2,094 bp. **b** Amplification of the region 1,000 bp up- and downstream of the *sigF* gene using primers BG 3990 and 3991. The wild-type genotype results in a product of 3,040 bp, whereas the complete deletion of the *sigF* gene is confirmed by a shift of the product to 2278 bp. **c** Amplification of the region 1,000 bp up- and downstream of the *pdhA* gene using primers BG 4563 and 4564. The wild-type genotype results in a product of 3,390 bp, whereas the complete deletion of the *pdhA* gene is confirmed by a shift of the product to 2,280 bp.

*B. smithii* ET 138 can be transformed with *E. coli*-*Bacillus* shuttle vector pNW33n with an efficiency of 5 × 10^3^ colonies per µg DNA [15]. To obtain mutants in strain ET 138, we planned to use a protocol similar to that used for *Geobacillus thermoglucosidans* (recently renamed from *G. thermoglucosidasius* [16]), which applies pNW33n-derivatives as thermosensitive integration plasmid [17]. To create a markerless l-lactate dehydrogenase (*ldhL*) knockout strain from which the *ldhL* gene was entirely deleted, ~1,000 bp regions flanking the *ldhL* gene and including the start and stop codon were cloned and fused together in plasmid pNW33n. Double homologous recombination of this plasmid with the ET 138 chromosome will fuse the start and stop codons of the gene, thereby removing the entire gene in-frame without leaving any marker (Figure 1). *B. smithii* ET 138 was transformed with pWUR732 and colonies were transferred once at 55°C on LB2 plates containing chloramphenicol. Subsequent PCR analysis of 7 colonies already showed integration of the plasmid DNA without the temperature increase normally performed with thermosensitive integration systems [17]. A mixture of single crossover integrants via both upstream and downstream regions together with no-integration (either caused by replicating plasmids or randomly integrated plasmids) genotype was observed in one colony, one colony showed a mixture of downstream crossover and wild-type genotype, and five colonies showed no single crossovers but only wild-type genotype. Serial transfer of the colonies containing single crossovers in liquid medium combined with replica plating to identify double recombinants repeatedly resulted in only wild-type double crossover mutants. The mixed genotype persisted after several subculturings on plates containing 7 and 9 µg/mL chloramphenicol in an attempt to obtain pure genotypes. After four transfers, however, also a colony was found that contained a mixture of double crossover knockout genotype together with upstream single crossover and wild-type genotype. After this point, we added glycerol or acetate as carbon sources to allow for a metabolism with minimal impact of the *ldhL* deletion. After streaking this colony to an LB2 plate containing 10 g/L glycerol, colonies were obtained that had lost the wild-type genotype but contained a mixture of both single crossovers and a double crossover knockout genotypes. A pure double crossover knockout genotype was observed after two transfers on the more defined TVMY medium supplemented with acetate at 65°C, creating strain ET 138 Δ*ldhL* (Figure 2a).

Similar to strain ET 138, also type strain *B. smithii* DSM 4216^T^ is transformable with pNW33n, with efficiencies of around 2x10^2^ colonies per µg DNA [15]. After transformation of strain DSM 4216^T^ with *ldhL*-knockout construct pWUR733 and transfer of colonies to a new plate at 55°C, PCR on 30 colonies showed mixtures of wild-type genotype and both single crossovers for all tested colonies. Contrary to what was observed with strain ET 138, several subculturings on TVMY supplemented with acetate at temperatures varying between 55 and 65°C did not result in a pure mutant genotype for derivatives of DSM 4216^T^. However, after the substrate was changed from acetate to lactate, a pure double-crossover knockout colony was obtained after two transfers, creating strain DSM 4216^T^ Δ*ldhL* (Figure 2a).

### Establishment of a *lacZ*-counter-selection system

Purifying the mixtures of plasmid integrated into the *B. smithii* genome via single and double crossovers during the construction of the *ldhL*-mutant strains required laborious PCR-screening. To simplify the screening procedure, a counter-selection tool was desirable to select against plasmid presence. A *lacZ*-counter-selection method has been described for the Gram-negative mesophile *Paracoccus denitrificans*, which is based on toxicity of high X-gal concentrations in the presence of β-galactosidase activity encoded by a *lacZ*-gene on the integration plasmid [18]. In the genome sequence of *B. smithii* ET 138 (unpublished data) no *lacZ* gene could be identified and the strain did not form blue colonies on plates containing X-gal. The *lacZ* gene from *B. coagulans* under control of the *B. coagulans pta*-promoter [14] was cloned into pNW33n, creating plasmid pWUR734. Introduction of pWUR734 into *B. smithii* ET 138 resulted in blue colonies in the presence of 25 mg/L X-gal. When *B. smithii* ET 138 harbouring pWUR734 was grown in the presence of 100 mg/L X-gal, the blue colonies were significantly smaller than on 25 mg/L, indicating toxicity of the X-gal cleavage product at high X-gal-concentrations (Additional file 3). To test the *lacZ*-counter-selection system, we chose *sigF* (*spoIIAC*) as the first knockout target gene, which is involved in the onset of *Bacillus* sporulation [19] (Figure 1). Transformation of strain ET 138 Δ*ldhL* with *sigF*-knockout vector pWUR735 containing the ~1,000 bp flanking regions of *sigF* yielded only blue and no white colonies, indicating functional expression of the *B. coagulanspta::lacZ* construct. Colony PCR on 8 colonies showed a mixture of single crossovers, wild-type and double-crossover knockout genotypes for six colonies and no single crossover but only double-crossover knockout and wild-type genotypes for two colonies. The latter two colonies, however, failed to grow after transfer to new LB2 plates. To obtain pure knockout strains, the counter-selection was applied by growing the colonies containing the mixed genotype of single and double crossovers overnight in 10 mL LB2 at 55°C, after which dilution series were plated on LB2 supplemented with 100 mg/L X-gal. A mix of large white (1–3 mm) and small blue (≤1 mm) colonies was obtained for all six cultures (Additional file 3). Colony PCR on three white colonies from one of the cultures showed the presence of one pure knockout, one pure wild-type and one mix of wild-type and single-crossover genotypes. The colony showing the pure knockout genotype was inoculated into liquid LB2, after which DNA was isolated and PCR analysis confirmed the knockout genotype and absence of plasmid, creating strain ET 138 Δ*ldhL* Δ*sigF* (Figure 2b).

### Construction of markerless triple mutant

To evaluate whether the *lacZ*-counter-selection method can be used repeatedly to delete multiple genes and to evaluate acetate production pathways in *B. smithii* ET 138, the α-subunit of the E1 component of the pyruvate dehydrogenase complex *pdhA* was targeted for deletion in strain ET 138 Δ*ldhL* Δ*sigF* using the *lacZ*-counter-selection system. Based on genome analysis, pyruvate dehydrogenase appears to be the only route to acetyl-CoA in *B. smithii* (unpublished data). Therefore, the mutant strain was expected to be dependent on acetate to form acetyl-CoA and the medium was supplemented with acetate at all times after transformation. After transformation with *pdhA*-knockout vector pWUR737 containing the ~1,000 bp flanking regions of *pdhA*, cells were plated on TVMY supplemented with acetate and chloramphenicol. PCR on 15 colonies showed 13 colonies with a mixture of wild-type genotype together with both single crossovers, one colony with a mixture of wild-type and downstream crossover and 1 colony with a pure downstream crossover. After one transfer of the downstream crossover colony on LB2 medium supplemented with acetate without chloramphenicol, a colony was picked showing a mixture of downstream crossover, wild-type and double crossover knockout genotypes. This colony was subjected to the counter-selection protocol by plating on 100 mg/L X-gal after overnight growth in liquid LB2, resulting in a mixture of small blue and large white colonies. From the 32 white colonies tested in PCR, 12 still showed single crossovers, 16 returned to wild-type and 4 showed a clean double crossover knockout genotype, creating triple mutant ET 138 Δ*ldhL* Δ*sigF* Δ*pdhA* (Figure 2c).

### Confirmation of sporulation deficiency

**Figure 3:**
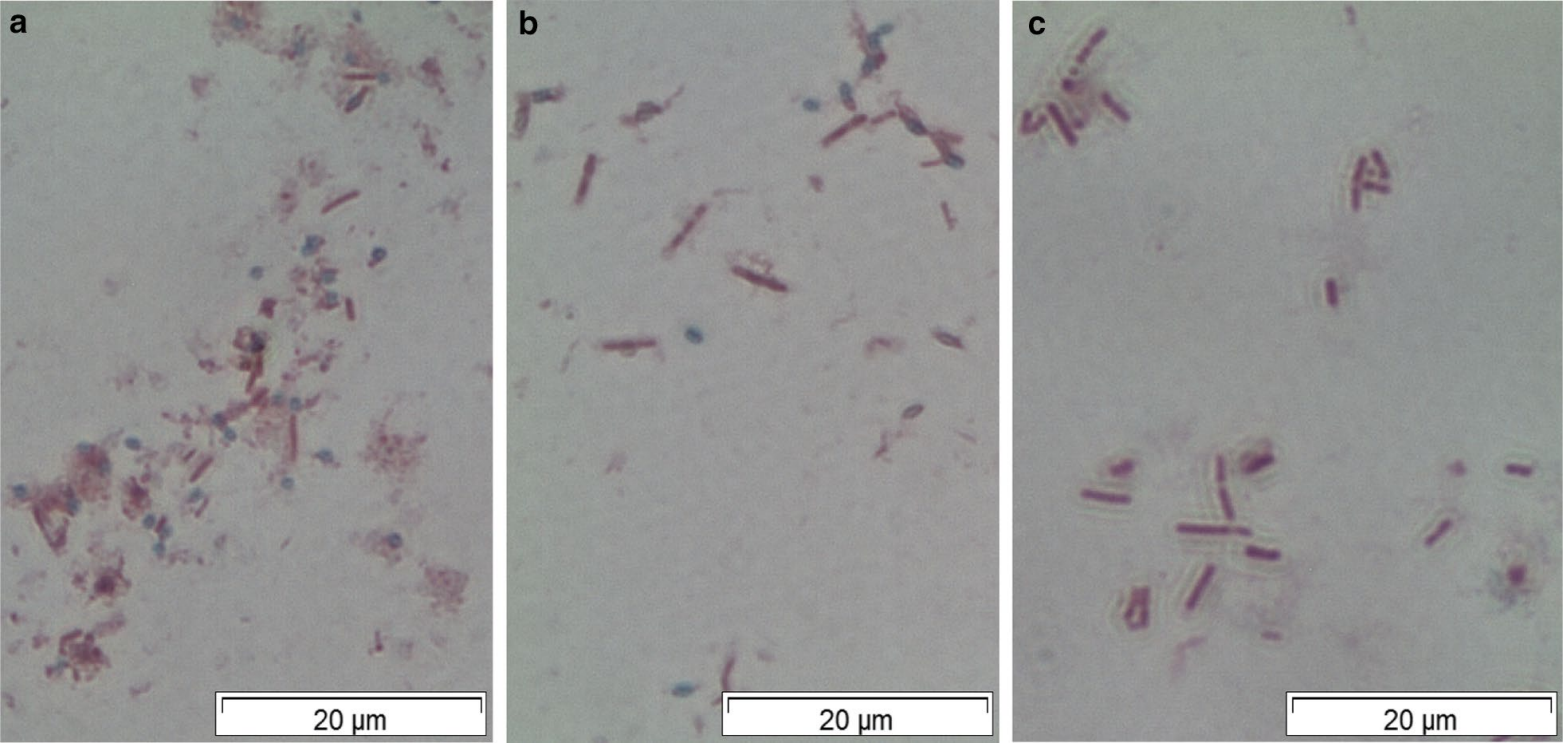
Schaeffer-Fulton staining on ET 138 wild-type and mutants. Cultures used were grown aerobically overnight in LB2 medium at 55°C and subsequently kept at room temperature for 24 h, after which the staining was performed. Pink-stained cells indicate intact cells, whereas spores are *green–blue*. **a** strain ET 138 wild-type, in which sporulation is observed. **b** strain ET 138 Δ*ldhL*, in which sporulation is observed. **c** Strain ET 138 Δ*ldhL* Δ*sigF*, where no sporulation is observed due to removal of the *sigF* gene.

To confirm that strain ET 138 Δ*ldhL* Δ*sigF* was unable to form spores, a Schaeffer-Fulton staining was performed (Figure 3). In the wild-type and the *ldhL*-mutant (Figure 3a, b) many spores were observed as indicated by the presence of green spheres, whereas no spores were observed in the *ldhL*-*sigF*-double mutant (Figure 3c). Pasteurisation of cultures of the wild-type and the Δ*ldhL*-strain resulted in colony counts of >5 × 10^5^ and 3 × 10^5^ per mL of cells, respectively. As expected, no colonies were observed after Pasteurisation of a culture of the Δ*ldhL* Δ*sigF* double mutant, while colony counts for the control treatment were >5 × 10^5^ per mL of cells for all three strains. Both assays confirm that the removal of the *sigF* gene results in a sporulation-deficient phenotype.

### Growth and production characteristics of mutant strains

To evaluate growth and fermentation characteristics of the mutant strains, both strains ET 138 and DSM 4216^T^ and all derived mutants were grown in tubes for 24 h under micro-aerobic conditions (Table 1). Whereas both ET 138 and DSM 4216^T^ wild-type strains produced mainly l-lactate (±95.5% of total products), the deletion of *ldhL* reduced l-lactate production to values around or below the detection limit, as shown by HPLC analysis combined with d- and l-lactate-specific enzyme assays (Table 1). For both strains, the main product shifted from l-lactate to acetate, with minor amounts of d-lactate, malate and succinate and increased concentrations of pyruvate compared to the wild-types. Both the OD_600_ and the final product titre of the mutants were about half that of the wild-types. Strain ET 138 Δ*ldhL* Δ*sigF* did not show significant differences compared to strain ET 138 Δ*ldhL* (Table 1). When the volume was increased from 25 to 40 mL in 50 mL tubes to further decrease the amount of oxygen present, the mutant strains showed even further reduced growth and production compared to the wild-type (data not shown). Similar results were obtained in 1 L pH controlled reactors (Additional file 4). Complementation of strain ET 138 Δ*ldhL* Δ*sigF* with its native *ldhL* gene and promoter expressed from pNW33n restored growth and l-lactate production to around wild-type levels (Table 1). Triple mutant ET 138 Δ*ldhL* Δ*sigF* Δ*pdhA* was unable to grow without acetate supplementation and produced mainly d-lactate and pyruvate. In this strain, very minor amounts of l-lactate (0.39 ± 0.02 mM) were observed. The final OD_600_ of this strain was on average comparable to the single and double mutant, but it showed less variation (Table 1).

**Table 1:** HPLC analysis of *B. smithii* ET 138 and DSM 4216^T^ wild-type and mutant strains

Strain	Products (mM) and measurement method							
	Enzymatic		HPLC					
	l-lac	d-lac	Lac	Ace	Pyr	Mal	Suc	OD_600_
DSM 4216 wild-type	15.85 ± *2.22*	0.73 ± *0.08*	20.67 ± *1.25*	9.12 ± *0.49*	0.21 ± *0.11*	0.06 ± *0.02*	0.27 ± *0.10*	0.785 ± *0.209*
DSM 4216 Δ*ldhL*	0.02 ± *0.03*	4.11 ± *0.86*	4.95 ± *3.35*	12.40 ± *3.16*	1.48 ± *1.14*	0.35 ± *0.07*	0.57 ± *0.22*	0.466 ± *0.057*
ET 138 wild-type	20.55 ± *4.09*	0.87 ± *0.05*	26.18 ± *7.66*	8.25 ± *2.59*	0.78 ± *0.31*	0.05 ± *0.08*	0.61 ± *0.16*	1.036 ± *0.136*
ET 138 Δ*ldhL*	0.08 ± *0.09*	1.08 ± *0.32*	1.73 ± *0.48*	14.33 ± *5.54*	0.76 ± *0.82*	0.33 ± *0.15*	0.72 ± *0.22*	0.581 ± *0.289*
ET 138 Δ*ldhL* Δ*sigF*	0.06 ± *0.05*	0.95 ± *0.20*	1.58 ± *0.62*	10.97 ± *2.13*	0.70 ± *0.41*	0.27 ± *0.08*	0.60 ± *0.16*	0.672 ± *0.102*
ET 138 Δ*ldhL* Δ*sigF* Δ*pdhA* ^a^	0.39 ± *0.02*	11.17 ± *0.47*	11.50 ± *2.07*	−3.19^a^ ± *1.24*	8.26 ± *0.82*	1.12 ± *0.14*	1.85 ± *0.37*	0.696 ± *0.119*
ET 138 Δ*ldhL* Δ*sigF* + pWUR736	18.13 ± 3.82	1.11 ± *0.40*	18.99 ± *5.19*	7.32 ± *1.33*	0.24 ± *0.16*	0.08 ± *0.18*	0.39 ± *0.17*	0.831 ± *0.200*
ET 138 Δ*ldhL* Δ*sigF* + pNW33n	0.14 ± *0.04*	2.57 ± *1.30*	3.17 ± *1.13*	10.14 ± *0.91*	0.94 ± *0.53*	0.07 ± *0.12*	0.36 ± *0.08*	0.535 ± *0.062*
ET 138 wild-type + pNW33n	22.12 ± *3.69*	0.69 ± *0.10*	21.42 ± *1.51*	5.63 ± *1.15*	0.65 ± *0.07*	0.05 ± *0.12*	0.34 ± *0.14*	0.849 ± *0.076*

Strains were grown in 25 mL TVMY supplemented with 10 g/L glucose in 50 mL Greiner tubes at 55°C for 24 h after transfer from a 10 mL-overnight culture. d- and l-lactate were distinguished via enzymatic assays, for which the lowest detection limit was 0.04 mM. The values shown are the results of three to fourteen independent experiments; numbers in italics are standard deviations.

*l* *-lac* l-lactate, *d* *-lac* d-lactate, *Ace* acetate, *Pyr* pyruvate, *Mal* malate, *Suc* succinate.

^a^These cultures were supplemented with 3 g/L ammonium acetate.

## Discussion

In this study, we developed an integration and counter-selection system for markerless and consecutive gene deletion in thermophilic *B. smithii*. As l-lactate is the main fermentation product of *B. smithii*, the *ldhL*-gene was selected as the first knockout target in order to use this bacterium for the production of other products. Wang et al. reported a laborious screening procedure when constructing a *B. coagulans* Δ*ldhL* strain and indicated that only 1 in 5,000 colonies showed a knockout genotype after double crossover [13]. We observed a similar bias in *B. smithii* for the second crossover to result only in wild-type revertants. Furthermore, in single colonies we observed mixtures of either upstream or downstream single crossovers, wild-type and double crossover knockout genotype, even after several transfers targeted at purifying the colony. These mixed genotypes were not only observed for the *ldhL* deletion, but also for the *sigF* and *pdhA* deletions. For *E. coli*, such mixed genotypes have been described to occur during recombineering with linear DNA fragments, where it has been attributed to polyploidy, *i.e.* the existence of multiple chromosomes [20]. The copy number of the genome of our organism is currently unknown, but in general this number is likely to be higher when cells are grown in rich medium compared to minimal medium [20]. Transformation attempts with *B. smithii* grown on minimal medium were not successful (data not shown), but when cells were grown on minimal medium after crossovers had occurred, the purification of pure genotypes was relatively fast and easy. Switching to minimal medium to overcome mixed genotype issues might be a useful approach for other species as well.

In other thermophilic *Bacilli*, integration events were reported after the growth temperature had been increased [17, 21]. Integration of the pNW33n-derived knockout plasmids in *B. smithii* without increasing the temperature indicates either a highly efficient recombination machinery, or plasmid instability already at 55°C, although this temperature is regarded as permissive for pNW33n [17]. In both *B. smithii* strains, we observed very stable plasmid integration into the genome via single crossover recombination in colonies grown on plates without antibiotic pressure. As this integrational stability hampered purification of pure double crossover knockouts, a counter-selection method was developed to select against plasmid presence. Most frequently used counter-selection systems are based on either auxotrophy or antibiotic resistance. The *lacZ*-system is fundamentally different in enabling clean gene deletions and re-use of the marker without inducing auxotrophies, as has been demonstrated in the mesophilic α-proteobacterium *Paracoccus denitrificans* [18]. The system can readily be used without making any prior gene deletions if the target strain does not possess β-glycosidase activity, as is the case in *B. smithii*. The system is based on the formation of toxic concentrations of inodxyl derivatives such as 5-bromo-4-chloro-3-indol, which is the cleavage product of 5-bromo-4-chloro-3-indolyl-β-D-galactopyranoside (X-Gal). Recently, indoxyl derivatives were shown to inhibit the growth of a wide variety of species growing at different temperatures, indicating that this system might be more widely applicable [22].

The *lacZ*-counter selection system considerably simplified the purification of single genotypes and enabled rapid and clean deletion of the *sigF* gene of ET 138 Δ*ldhL* to create a sporulation-deficient strain. The system can be used repetitively to make multiple sequential deletions in the same strain as shown by the generated triple mutant ET 138 Δ*ldhL* Δ*sigF* Δ*pdhA*. For both the *sigF* and the *pdhA* deletion, around 33% of the tested colonies were false positive after counter-selection, as they were white while still having a single crossover, which might be due to spontaneous disruption of the *lac*Z coding sequence or its promoter. Even with 33% false-positive white colonies, the screening for mutants is significantly simplified when using the *lacZ*-counter-selection system. A drawback of the system is that it does not force the double crossover in the direction of the knockout and can result in wild-type revertants. Whenever possible, cultivation conditions should be chosen such that the knockout cells will not have a large disadvantage over the wild-type cells. During this study, culturing in liquid medium resulted only in wild-type revertants, whereas knockouts were successfully obtained when the cultures were kept on plates. Supplementation of the plates with the gluconeogenic substrates acetate or lactate might have further reduced the disadvantage of knockouts over wild-types.

Removal of the *sigF* gene from *B. smithii* ET 138 resulted in a sporulation-deficient strain. Sporulation deficiency is desired in industrial settings both for practical and safety reasons. *B. smithii* was shown to have highly thermo-resistant spores [23, 24] and was found to be the most dominant species together with *Geobacillus pallidus* (recently renamed to *Aeribacillus pallidus* [16]) as highly thermostable spores in food. Both species were found to be non-cytotoxic [23]. The *sigF*-gene has been targeted successfully to create sporulation-deficient strains in several other *Bacilli* as well as in *Clostridia*, such as in *B. coagulans* [14], *B. licheniformis* [25, 26], *B. subtilis* [19] and *Clostridium acetobutylicum* [27].

Removal of the *ldhL*-gene from *B. smithii* ET 138 and DSM 4216^T^ eliminated l-lactate production in both strains to values around or below the detection limit, which is similar to an *ldhL*-knockout of *B. coagulans* [13], a close relative of *B. smithii*. A lactate racemase was not found in the ET 138 genome, but the methylglyoxal pathway was annotated towards both d- and l-lactate (unpublished data) and this pathway is most likely the origin of the trace amounts of lactate observed in triple mutant ET 138 Δ*ldhL* Δ*sigF* Δ*pdhA*. Products such as acetoin, 2,3-butanediol, formate and ethanol have not been detected in any of the *B. smithii* mutant strains, while these were observed in *B. coagulans* Δ*ldhL* [13]. The absence of these products is in line with the absence of the acetolactate decarboxylase and pyruvate-formate lyase genes from the *B. smithii* genomes (unpublished data). The *B. smithii ldhL*-mutants produced mainly acetate and some d-lactate and showed reduced growth and acid production under micro-aerobic conditions, which was restored by plasmid-based complementation in strain ET 138 Δ*ldhL* Δ*sigF*. The deficiency in anaerobic capacities is likely to be caused by the redox imbalance that results from the elimination of its main NAD^+^-regeneration pathway and the apparent lack of an alternative NAD^+^-regeneration pathway, such as that to 2,3-butanediol. The lack of these pathways potentially makes *B. smithii* an interesting platform organism as only the *ldhL* gene needs to be removed in order to eliminate production, after which the desired product pathways can be inserted.

Acetate was the main fermentation product of the *B. smithii* strains lacking the *ldhL* gene, but the standard pathway to acetate via acetate kinase and phosphotransacetylase as well as a pyruvate-formate lyase gene are absent in the genomes of both strains ET 138 and DSM 4216^T^ (unpublished data). To remove acetate production, the *pdhA* gene encoding the α-subunit of the E1 component of the pyruvate dehydrogenase complex was removed from strain ET 138 Δ*ldhL* Δ*sigF*. The resulting strain ET 138 Δ*ldhL* Δ*sigF* Δ*pdhA* did not produce acetate and was unable to grow without acetate supplementation. This implies that pyruvate dehydrogenase is the main enzyme responsible for pyruvate to acetyl-CoA, which is in line with the absence of a *pfl* gene. Acetate utilization was previously suggested as a rescue pathway for redox balance in *Lactococcus lactis* Δ*ldhL* [28]. We tested acetate supplementation of strain ET 138 Δ*ldhL* Δ*sigF* but this did not improve growth or production (data not shown).

## Conclusion

In this study, we established a clean gene deletion system for *B. smithii* using *lacZ* counter-selection. We constructed *ldhL* mutants in the type strain *B. smithii* DSM 4216^T^ and compost isolate strain ET 138. In the latter strain, triple mutant Δ*ldhL* Δ*sigF* Δ*pdhA* was constructed, which does not produce l-lactate and acetate and is no longer capable of forming spores. Although further studies and modifications are needed to restore anaerobic growth and production capacities, the *lacZ*-counter-selection system combined with mutant-specific culturing strategies provides a tool for the construction of markerless gene deletions in thermophilic *B. smithii*. This enables the development of this species into a platform organism and provides tools for studying its metabolism, which appears to be different from its close relatives such as *B. coagulans* and other *Bacilli*.

## Methods

### Bacterial strains and growth conditions

Strains used in this study are listed in Table 2. All *B. smithii* strains were routinely cultured at 55°C unless stated otherwise. *E. coli* DH5α was grown at 37°C. For growth experiments with strain ET 138 Δ*ldhL* Δ*sigF* Δ*pdhA*, 3 g/L ammonium acetate was added to all media at all times. For all tube and plate cultures, carbon substrates were used in a concentration of 10 g/L unless stated otherwise. Substrates and acetate were added separately as 50% autoclaved solutions after autoclavation of the medium. For plates, 5 g/L Gelrite (Roth) was added. Unless indicated otherwise, chloramphenicol was added in concentrations of 25 µg/mL for *E. coli* and 7 µg/mL for *B. smithii*.

**Table 2:** *B. smithii* strains used in this study

Strain	Description	Reference/origin
DSM 4216^T^	Wild-type, type strain of the species	DSMZ
DSM 4216^T^ Δ*ldhL*	DSM 4216^T^ with clean *ldhL*-deletion	This study
ET 138	Wild-type, natural isolate	[15]
ET 138 Δ*ldhL*	ET 138 with clean *ldhL*-deletion	This study
ET 138 Δ*ldhL* Δ*sigF*	ET 138 Δ*ldhL* with clean *sigF*-deletion	This study
ET 138 Δ*ldhL* Δ*sigF* Δ*pdhA*	ET 138 Δ*ldhL* Δ*sigF* with clean *pdhA*-deletion	This study

*DSMZ* Deutsche Sammlung von Microorganismen und Zellkulturen, Germany.

Thermophile Vitamin Medium with Yeast extract (TVMY) contained per L: 8.37 g MOPS; 0.5 g yeast extract (Roth), 100 mL 10× concentrated Eight Salt Solution (ESS), 1 mL filter sterile 1,000× concentrated vitamin solution, and 1 mL filter sterile 1,000× concentrated metal mix. ESS contained per L: 2.3 g K_2_HPO_4_; 5.1 g NH_4_Cl; 50 g NaCl; 14.7 g Na_2_SO_4_; 0.8 g NaHCO_3_; 2.5 g KCl; 18.7 g MgCl_2_.6H_2_O; 4.1 g CaCl_2_.2H_2_O). 1,000× concentrated metal mix contained per L: 16.0 g MnCl_2_.6H_2_O; 1.0 g ZnSO_4_; 2.0 g H_3_BO_3_; 0.1 g CuSO_4_.5H_2_O; 0.1 g Na_2_MoO_4_.2H_2_0; 1.0 g CoCl_2_.6H_2_O; 7.0 g FeSO_4_.7H_2_O. 1,000× concentrated vitamin mix contained per L: 0.1 g thiamine; 0.1 g riboflavin; 0.5 g nicotinic acid; 0.1 g panthothenic acid; 0.5 g pyridoxamine, HCl; 0.5 g pyridoxal, HCl; 0.1 g D-biotin; 0.1 g folic acid; 0.1 g *p*-aminobenzoic acid; 0.1 g cobalamin. The pH of TVMY was set to 6.94 at room temperature and the medium was autoclaved for 20 min at 121°C, after which vitamin solution, metal mix and substrate were added.

LB2 medium contained per L: 10 g tryptone (Oxoid), 5 g yeast extract (Roth), 100 mL ESS. The pH was set to 6.95 at room temperature and the medium was autoclaved for 20 min at 121°C. For all mutant strains, vitamins and metals as described above for TVMY were also added to LB2.

To evaluate product profiles and growth, cells were inoculated from glycerol stock into 10 mL TVMY supplemented with 10 g/L glucose in a 50 mL Greiner tube and grown overnight at 55°C and 150 rpm. Next morning, 250 µL cells was transferred to 25 mL of the same medium in 50 mL Greiner tubes and incubated at 55°C and 150 rpm for 24 h, after which OD_600_ was measured and fermentation products were analysed.

### Plasmid construction

Plasmids and primers used in this study are shown in Tables 3 and 4. Genomic DNA from *B. smithii* strains was isolated using the MasterPure™ Gram Positive DNA Purification Kit (Epicentre). *E. coli* DH5α heat shock transformation was performed according to standard procedures [29]. All restriction enzymes and polymerases were obtained from Thermo Scientific. PCR products were gel-purified from a 0.8% agarose gel using the Zymoclean™ Gel DNA Recovery Kit.

**Table 3:** Plasmids used in this study

Plasmid	Description	Reference/origin
pNW33n	*E. coli*-*Bacillus* shuttle vector, cloning vector, Cm^R^	BGSC
pWUR732	*ldhL*-KO vector for ET138: pNW33n + *ldhL*-flanks	This study
pWUR733	*ldhL*-KO vector for DSM 4216: pNW33n + *ldhL*-flanks	This study
pWUR734	pNW33n + *B. coagulans* P_*pta*_-*lacZ*	[14]; this study
pWUR735	*sigF*-KO vector for ET138: pWUR734 + ET 138 *sigF*-flanks	This study
pWUR736	*ldhL*-restoration vector for ET138: pNW33n + *ldhL*-gene from ET 138 under its native promoter (525 bp us of *ldhL*)	This study
pWUR737	*pdhA*-KO vector for ET138: pWUR734 + ET 138 *pdhA* -flanks	This study

*Cm* ^*R*^ chloramphenicol resistance gene, *KO* knockout, *BGSC* Bacillus Genetic Stock Centre, USA, *us* upstream, *bp* base pairs.

**Table 4:** Primers used in this study

BG nr	Sequence 5′–3′	Purpose
3464	AACTCTCCGTCGCTATTGTAACCA	Check plasmid presence
3465	TATGCGTGCAACGGAAGTGAC	Check plasmid presence
3633	GCCGTCGACCATTTGCAGTAGGTCTCGATC	*ldhL*-us-Fw
3636	GCCGAATTCTAGGTCACCAAAGACGAAATTG	*ldhL*-ds-Rv
3637	GCTCCCTTTGTATGGTCGTTTACATAATAAGAAACTCCTTTCGTCATTTC	*ldhL*-us-Rv
3638	GAAATGACGAAAGGAGTTTCTTATTATGTAAACGACCATACAAAGGGAGC	*ldhL*-ds-Fw
3664	AGGGCTCGCCTTTGGGAAG	Int. check, in plasmid
3663	ATCGCGTGAAATGTTCTAATGG	Int. check *ldhL* on chr.-Fw
3669	AACCGATGCCGTTGATTAAAG	Int. check *ldhL* on chr.-Rv
3887	GCCGAGCTCTTGCCGGAATTCTTTCAC	*pta*-*lacZ*-Fw
3888	GCCTCATGACTATTTTTCAATTACCTGCAAAATTTTC	*pta*-*lacZ*-Rv
3971	GCCGAATTCAGCTAATCTTGTTGACGGTTTTC	*sigF*-ds-Fw
3972	GTAACTAAGGAGTCGTGCCTTAACGATTCATGTGCTTTTTTTTG	*sigF*-ds-Rv
3973	CAAAAAAAAGCACATGAATCGTTAAGGCACGACTCCTTAGTTAC	*sigF*-us-Fw
3974	GCCGTCGACCTCTGATTTAGAAGATGGAGGTTTT	*sigF*-us-Rv
3990	CGCCTATTCTTTTCGCTAAAATCGG	Int. check *sigF* on chr.-Fw
3991	ATAAGCTGCAGAGGGATATACAC	Int. check *sigF* on chr.-Rv
4534	GCCTCTAGAATTGGTCATTTGATTAGA	ET 138 *ldhL* + prom.-Fw
4535	GCCAAGCTTTTAAGAAAGTACTTTATT	ET 138 *ldhL* + prom.-Rv
4522	GCCGAATTCGAGGTACATAGCCCGGAATC	*pdhA*-us-Fw
4523	GTCATTTGCGGCATGGCTTACATTCGTGTCACCTCTTCCTTTC	*pdhA*-us-Rv
4524	GAAAGGAAGAGGTGACACGAATGTAAGCCATGCCGCAAATGAC	*pdhA*-ds-Fw
4525	GCCGTCGACCATCCTCATAACGGCCATCC	*pdhA*-ds-Rv
4563	GTTTCACATACCATTTAACGATTT	Int. check *pdhc* on chr.-Rv
4564	GTCAATAGGTGCAAATGGATTTTC	Int. check *pdhc* on chr.-Fw

*us* upstream flanking region, *ds* downstream flanking region, *Fw* forward primer, *Rv* reverse primer, *Int.* integration, *chr.* chromosome, *prom.* promoter.

For the construction of ET 138 *ldhL*-knockout vector pWUR732, the flanking regions of the *ldhL* gene were PCR-amplified from genomic DNA using primers BG3633 and BG3637 (upstream, 923 bp) and BG3638 and BG3636 (downstream, 928 bp) using Phusion polymerase. DSM 4216^T^*ldhL*-knockout vector pWUR733 was created using the same primers, resulting in a 913 bp upstream region and 935 downstream. After gel-purification, an overlap extension PCR was performed in which the upstream and downstream region were fused using primers BG3633 and BG3636, making use of the complementary overhang in primers BG3637 and BG3638. The resulting PCR product was again gel-purified and subsequently cut with EcoRI and SalI using the restriction sites included in primers BG3633 and BG3636, as was plasmid pNW33n. After restriction, the fusion product and pNW33n were ligated using T4 ligase (Thermo Scientific) for 1 h at room temperature and transformed to heat shock competent *E. coli* DH5α.

To create *lacZ*-containing plasmid pWUR734, primers BG3887 and BG3888 were used to generate the *B. coagulans* P_*pta*_-*lacZ* promoter-gene fusion fragment by PCR using plasmid pPTA-LAC as template [14]. The resulting fragment was cloned into pNW33n using SacI and BspHI and transformed to heat shock competent *E. coli* DH5α.

The ET 138 *sigF* flanking regions fragments of the knockout-plasmid pWUR735 were generated by using primers BG3971 and BG3972 (downstream, 970 bp) and BG3973 and BG3974 (upstream, 976 bp). The flanks were fused by PCR using primers BG3971 and BG3974, cloned into pWUR734 using EcoRI and SalI and transformed to heat shock competent *E. coli* DH5α. Plasmid pWUR737 containing the *pdhA* flanks in pWUR734 was constructed in a similar manner, using primers BG4522 and BG4523 to generate the *pdhA* upstream flank (1011 bp) and BG4524 and BG4525 to generate the *pdhA* downstream flank (1037 bp) by PCR. The flanks were fused by PCR using primers BG4522 and 4525 and cloned into pWUR737 using EcoRI and SalI.

For construction of ET 138 *ldhL*-complementation plasmid pWUR736, the *ldhL* gene with its native promotor (until 525 bp upstream of the gene) was amplified from the ET 138 genome using primers BG4534 and BG4535. The fragment was cloned into pNW33n using HindIII and XbaI.

Transformed *E. coli* DH5α colonies were picked and inoculated into 5 mL LB containing 25 µg/mL chloramphenicol, after which plasmids were isolated using the GeneJET Plasmid Miniprep Kit (Thermo Scientific) and the integrity of the cloned fragments was confirmed by DNA sequencing (GATC, Germany). Plasmids for transforming ET 138 and DSM 4216^T^ were extracted from DH5α via maxiprep isolation (Genomed Jetstar 2.0).

### Competent cell preparation and electroporation of *B. smithii*

*B. smithii* was transformed by electroporation as described previously [15]. In brief, *B. smithii* cells were grown overnight at 55°C in 10 mL LB2 in a 50 mL Greiner tube and next morning diluted to an OD_600_ of 0.08 in 100 mL LB2 in a 500 mL baffled Erlenmeyer flask (DSM 4216^T^) or 1 L bottle (ET 138). Cells were grown to an OD_600_ between 0.45 and 0.65 and made competent as described previously [30]. Electroporation was performed applying settings of 2.0 kV, 25 µF and 400 Ω in a 2 mm cuvette for ET 138 and 1.5 kV, 25 µF and 600 Ω in a 1 mm cuvette for DSM4216^T^ (ECM 630 electroporator, GeneTronics Inc.). 2–5 µg plasmid DNA was added to the cells for electroporation and LB2 medium was used for recovery at 52°C for 3 h. After overnight growth on LB2 plates containing 7 µg/mL chloramphenicol and in the case of *lacZ*-containing plasmids also 20 µg/mL X-gal at 52°C, several colonies were streaked to a fresh plate and grown overnight at 55°C, after which colony PCR was performed to confirm the presence of the plasmid and check for integration.

### *B. smithii* colony PCR

Colony PCR on *B. smithii* was performed using the InstaGene Matrix protocol (BioRad) with several modifications: colonies were picked and resuspended in 200 µL MQ water in a 1.5 mL Eppendorf tube and centrifuged at 13,200 rpm for 2 min. The supernatant was removed, 100 µL InstaGene Matrix was added to the pellet and this was incubated at 55°C for 30 min. After this, the mixtures were vortexed at high speed for 10 s and incubated at 99°C in a heat block (Eppendorf) for 8 min, vortexed again for 10 s and centrifuged at 13200 for 3 min. Subsequently, 10 µL of the resulting supernatant was used per 25 µL PCR reaction and the remainder was stored at −20°C for later use.

### Construction of *B. smithii* ET 138 and DSM 4216^T^*ldhL* mutants

*B. smithii* ET 138 was made competent and electroporated with pWUR732. After transformation, colonies were subjected to colony PCR using primers BG3663 and BG3669 to distinguish double crossover wild-type and knockout genotypes, BG3664 and BG3669 to check chromosomal integration of the plasmid and BG3464 and BG3465 to check plasmid presence. A colony showing both upstream and downstream integration as well as wild-type genotype was picked and streaked to a fresh LB2 plate supplemented with 7 µg/mL chloramphenicol and grown overnight at 55°C, which was repeated one more time at 7 µg/mL chloramphenicol and then twice at 9 µg/mL chloramphenicol. From the last plate, a colony was picked that showed both wild-type and double-crossover knockout genome, as well as a single crossover via the upstream region. After overnight growth on LB2 supplemented with 7 µg/mL chloramphenicol and 1% glycerol at 55°C, a colony was picked that did no longer show the wild-type genotype. This colony was transferred twice on TVMY supplemented with 50 mM ammonium acetate at 65°C, resulting in a pure knockout genotype. Genomic DNA isolation was performed on liquid cultures grown overnight in TVMY containing 50 mM ammonium acetate to confirm the knockout genotype and lack of plasmid. The PCR product from primers BG3663 and BG3669 was purified (Zymo DNA Clean & Concentrator) to confirm correct deletion of the gene by sequencing.

*B. smithii* DSM4216^T^ was made competent and transformed with pWUR733, colonies were streaked to a new LB2 plate with 7 µg/mL chloramphenicol and checked for integrations as described for *B. smithii* ET 138. A colony showing wild-type genotype as well as both upstream and downstream integration was picked and streaked to a fresh LB2 plate supplemented with 9 µg/mL chloramphenicol and grown overnight at 55°C. Subsequently, it was transferred 3 more times on the same medium at 55°C and once at 65°C, after which 1 transfer was performed on LB2 containing 7 µg/mL chloramphenicol and 1% (v/v) glycerol at 55°C. Next, the colony was transferred several times on TVMY containing 50 mM ammonium acetate at 55°C and 65°C. During the whole procedure, colony PCR using the above-mentioned primers was performed and only colonies showing single crossover (combined with wild-type genotype) were transferred. Subsequently, a colony showing double crossover knockout genotype mixed with wild-type and single crossovers was purified by transferring to TVMY containing 50 mM ammonium acetate 5 more times at 60°C and then 2 times on TVMY containing 50 mM lactate. Genomic DNA isolation was performed on liquid cultures grown overnight in TVMY containing 50 mM lactate to confirm the knockout genotype and lack of plasmid. The PCR product from primers BG3663 and BG3669 was purified (Zymo DNA Clean & Concentrator) to confirm correct deletion of the gene by sequencing.

### Construction of *B. smithii* ET 138 Δ*ldhL* Δ*sigF* mutant using *lacZ* counter-selection

After transformation of *B. smithii* ET 138 Δ*ldhL* with plasmid pWUR735, blue colonies were transferred to new LB2 plates supplemented with 7 µg/mL chloramphenicol twice at 55°C. Subsequently, colony PCR was performed using primers BG3990 and BG3991 to distinguish double crossover wild-type and knockout genotypes, and primers BG3990 and BG3664 to check chromosomal integration of the plasmid and BG3464 and BG3465 to check plasmid presence. Several colonies showing a mixture of single crossovers, wild-type and double-crossover knockout genotype were transferred to 10 mL LB2 in 50 mL tubes and grown overnight at 55°C, after which dilution series were plated on LB2 supplemented with 100 µg/mL X-gal. After overnight growth, white colonies were picked and transferred twice for overnight growth at 55°C on LB2 plates, after which colony PCR was performed to distinguish wild-type from double-crossover knockout genotype. Genomic DNA isolation was performed on liquid culture grown overnight in LB2 to confirm the knockout genotype and lack of plasmid. The resulting PCR product was purified (Zymo DNA Clean & Concentrator) to confirm correct deletion of the gene by sequencing and glycerol stocks were made.

### Construction of *B. smithii* ET 138 Δ*ldhL* Δ*sigF* Δ*pdhA* mutant using *lacZ* counter-selection

*B. smithii* ET 138 Δ*ldhL* Δ*sigF* was transformed with plasmid pWUR737 and recovery and plating was performed at 52°C on TVMY containing 50 mM ammonium acetate, 7 µg/mL Cm and 20 µg/mL X-gal. Next day, blue colonies were streaked to new plates containing the same medium without X-gal and grown overnight, after which colony PCR was performed using primers BG4563 and BG4564 to distinguish double crossover wild-type and knockout genotypes and BG4564 and BG3664 to check chromosomal integration of the plasmid. Several colonies showing either single crossover and/or double crossover were transferred to fresh TVMY containing 50 mM ammonium acetate plates containing antibiotics and grown overnight at 52°C, after which the same colony PCR was performed on the new colonies. One colony that showed only downstream crossover, whereas the others showed only upstream crossover, did not grow any further on TVMY containing 50 mM ammonium acetate and thus was transferred to LB2 containing 50 mM ammonium acetate plates without antibiotics and subjected again to colony PCR after overnight growth. One colony showing a strong double crossover knockout band was transferred to LB2 containing 50 mM ammonium acetate and grown overnight, after which it was inoculated into 10 mL liquid LB2 containing 50 mM ammonium acetate in a 50 mL tube and grown overnight at 55°C and 150 rpm. Subsequently, dilution series were plated on LB2 containing 50 mM ammonium acetate supplemented with 100 µg/mL X-gal. After overnight growth, white colonies were streaked to a new plate LB2 containing 50 mM ammonium acetate and subjected to colony PCR. Several colonies showing pure knockout genotype were inoculated into 10 mL LB2 containing 50 mM ammonium acetate and grown overnight. Genomic DNA isolation was performed on liquid cultures grown overnight in LB2 to confirm the knockout genotype and lack of plasmid, after which the resulting PCR product was purified (Zymo DNA Clean & Concentrator) to confirm correct deletion of the gene by sequencing.

### Sporulation assays

For the Schaeffer-Fulton staining [31], *B. smithii* strains ET 138 wild-type, ET 138 Δ*ldhL* and ET 138 Δ*ldhL* Δ*sigF* were grown aerobically overnight in LB2 medium at 55°C and subsequently kept at room temperature for 24 h, after which a droplet of the cell culture was added to a microscopy slide and allowed to air-dry. The sample was heat-fixed above a gas flame and covered with a piece of absorbance paper, after which the slide was flooded with 50 g/L malachite green (4-[(4-dimethylaminophenyl)phenyl-methyl]-N,N-dimethylaniline) and heated to steam twice. The absorbance paper was removed and the slide was washed with tap water, after which it was flooded with 25 g/L safranin for 30 s, washed with tap water, dried with paper and evaluated under the microscope (Carl Zeiss Primo Star 1000x magnification with Olympus Soft Imaging Solutions Camera and analySIS 5.0 imaging software).

For the pasteurization assay, strains ET 138 wild-type, ET 138 Δ*ldhL* and ET 138 Δ*ldhL* Δ*sigF* were grown for 24 h in 10 mL LB2 in a 50 mL Greiner tube. 1 mL culture was transferred to a 1.5 mL reaction tube in duplicate, of which one tube was incubated at 60°C for 45 min as a control and one at 85°C for 45 min. After this, a 100× dilution was plated on LB2 and incubated overnight at 55°C, after which colonies were counted.

### Analytical methods

Sugar and fermentation products were quantified using a high-pressure liquid chromatography (HPLC) system (Thermo) equipped with a UV1000 detector operating on 210 nm and a RI-150 40°C refraction index detector and containing a Shodex RSpak KC-811cation-exchange column. The mobile phase consisted of 5 mM H_2_SO_4_ and the column was operated at 0.8 mL/min and 80°C. All samples were diluted 1:1 with 10 mM DMSO in 0.04 N H_2_SO_4_. l-lactate and d-lactate kits from Megazyme (K-LATE and K-DATE) were used to distinguish between l-lactate and d-lactate according to the manufacturer’s protocol.

## Additional files

Additional file 1: This file shows the original gel for Figure 2A and B. It is the same gel as in Figure C/additional file 2, but with a shorter exposure time. Additional file 2: This file shows the original gel for Figure 2 C. It is the same gel as in Figure A-B/additional file 1, but with a longer exposure time. Additional file 3:
*B. smithii* Δ*ldhL*-Δ*sigF* after counter-selection on plates containing 100 μg/mL X-gal. This figure shows the difference in colony size on 100 µg/mL X-gal after *lacZ* counter-selection. Additional file 4: Fermentation analysis of *B. smithii* ET 138 wild-type and mutant strains in 1 L pH-controlled bioreactors after ±24 h. This table shows fermentation data in pH-controlled lab-scale reactors to supplement the data in tubes described in the main text.
