# Supplementary material for: Establishment of markerless gene deletion tools in thermophilic Bacillus smithii and construction of multiple mutant strains
Source: Microb Cell Fact. 2015 Jul 7;14:99. doi: 10.1186/s12934-015-0286-5 (PMC4494709; doi:10.1186/s12934-015-0286-5)

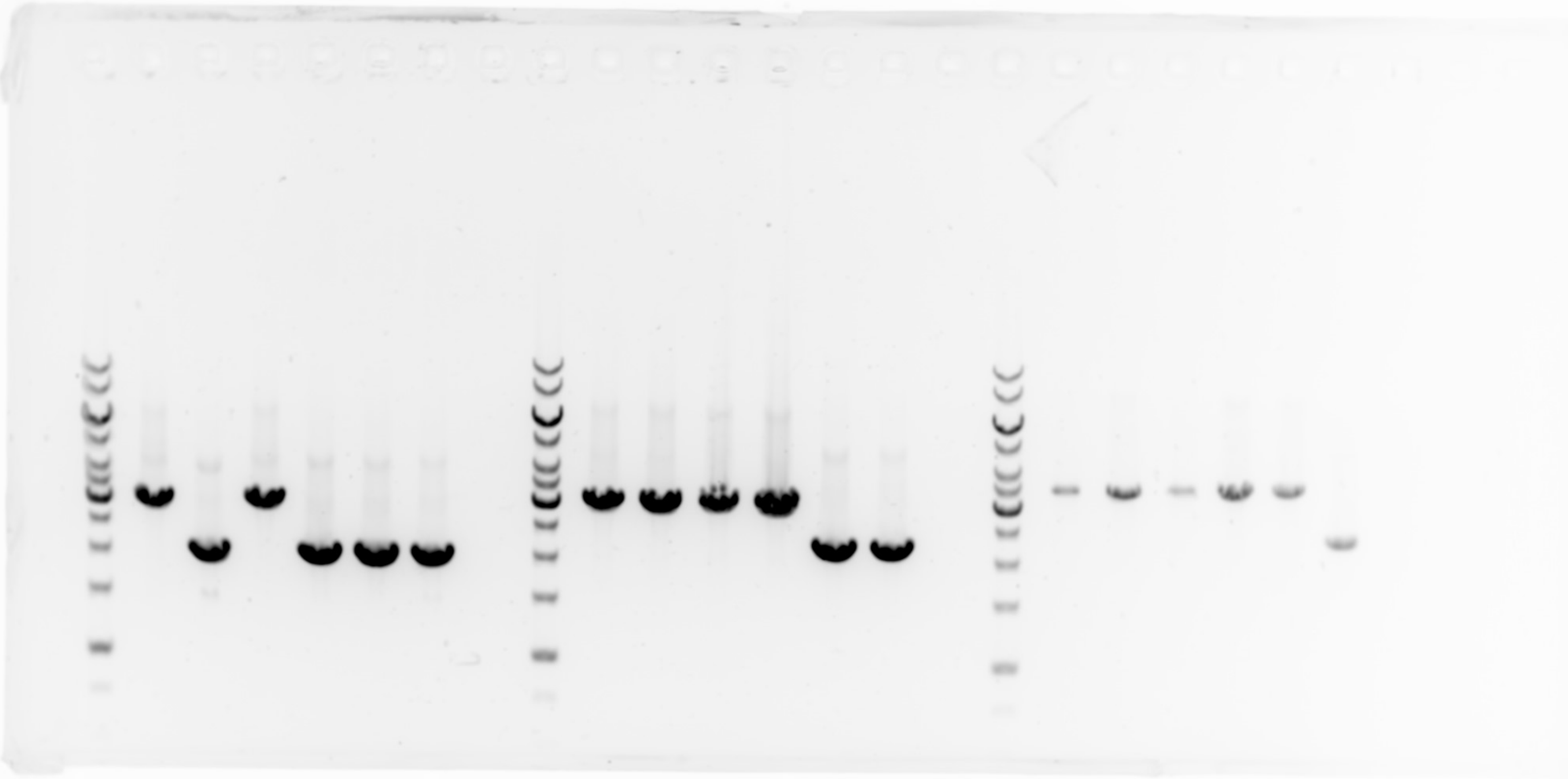

Supplement: Additional file 1: — This file shows the original gel for Figure 2A and B. It is the same gel as in Figure C/additional file 2, but with a shorter exposure time. [file 12934_2015_286_MOESM1_ESM.pdf]
