# Supplementary figures and images for: Establishment of markerless gene deletion tools in thermophilic Bacillus smithii and construction of multiple mutant strains
Source: Microb Cell Fact. 2015 Jul 7;14:99. doi: 10.1186/s12934-015-0286-5 (PMC4494709; doi:10.1186/s12934-015-0286-5)

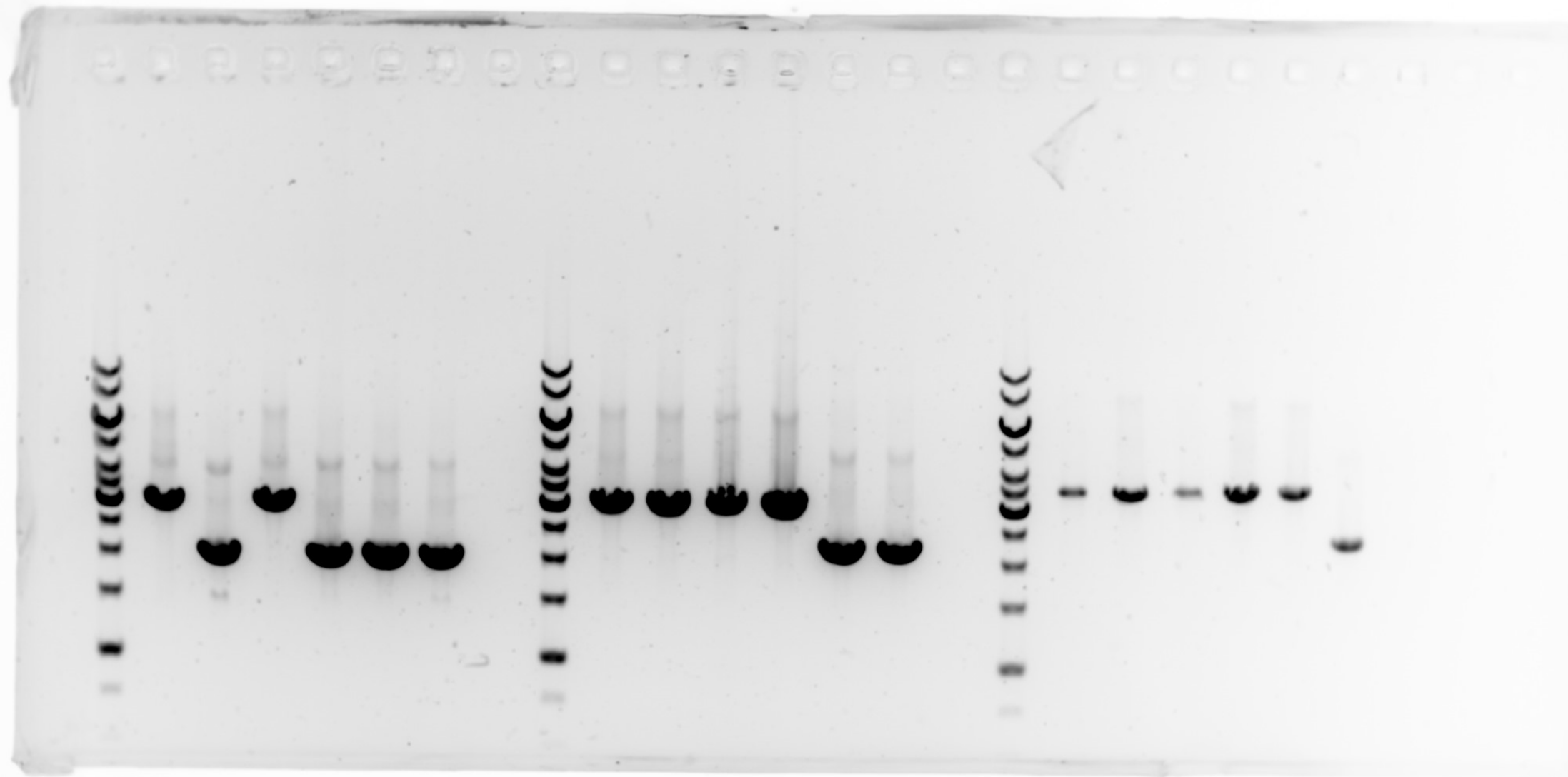

Supplement: Additional file 2: — This file shows the original gel for Figure 2 C. It is the same gel as in Figure A-B/additional file 1, but with a longer exposure time. [file 12934_2015_286_MOESM2_ESM.pdf]
