# Supplementary material for: Establishment of markerless gene deletion tools in thermophilic Bacillus smithii and construction of multiple mutant strains
Source: Microb Cell Fact. 2015 Jul 7;14:99. doi: 10.1186/s12934-015-0286-5 (PMC4494709; doi:10.1186/s12934-015-0286-5)

Additional file 3.

B. smithii Δ*ldhL*-Δ*sigF* after counter-selection on plates containing 100 µg/mL X-gal. Blue colonies still contain the plasmid-encoded *lacZ* gene and are smaller than the white colonies. White colonies have lost the plasmid and thus are not inhibited by the high concentration of the X-gal cleavage product.

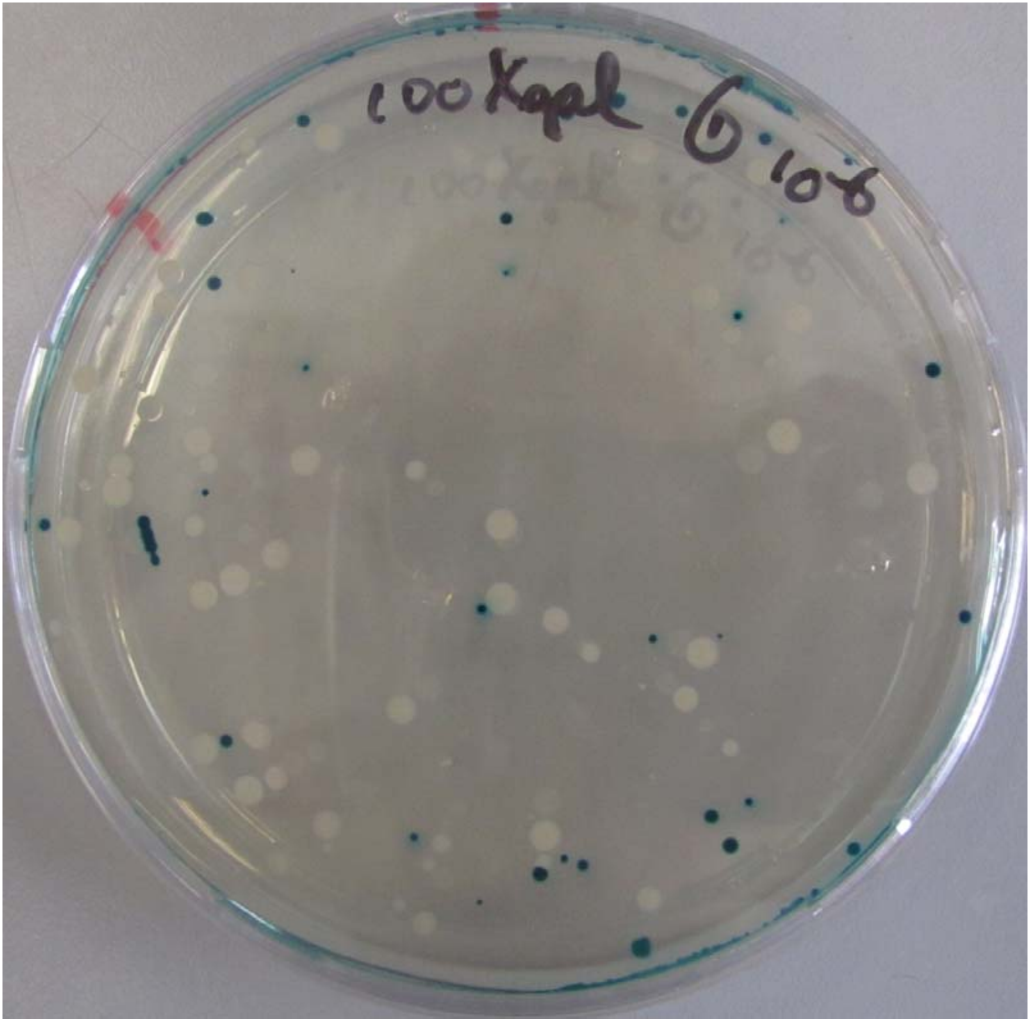

Supplement: Additional file 3: — B. smithii ΔldhL-ΔsigF after counter-selection on plates containing 100 μg/mL X-gal. This figure shows the difference in colony size on 100 µg/mL X-gal after lacZ counter-selection. [file 12934_2015_286_MOESM3_ESM.pdf]
