# Supplementary material for: Establishment of markerless gene deletion tools in thermophilic Bacillus smithii and construction of multiple mutant strains
Source: Microb Cell Fact. 2015 Jul 7;14:99. doi: 10.1186/s12934-015-0286-5 (PMC4494709; doi:10.1186/s12934-015-0286-5)
Supplement: Additional file 4: — Fermentation analysis of B. smithii ET 138 wild-type and mutant strains in 1 L pH-controlled bioreactors after ±24 h. This table shows fermentation data in pH-controlled lab-scale reactors to supplement the data in tubes described in the main text. [file 12934_2015_286_MOESM4_ESM.pdf]

Additional file 4.

Fermentation analysis of *B. smithii* ET 138 wild-type and mutant strains in 1 L pH-controlled bioreactors after ±24 h.

	Products (mM)[1]						
**ET 138 strain**	**Lac**	**Ace**	**Pyr**	**Mal**	**Suc**	**Total**	**OD_{600}**
wild-type	73.14	3.97	0.34	2.67	0.34	80.46	0.891
Δ*ldhL*	1.82	3.72	3.15	1.19	0.12	10.01	0.251
Δ*ldhL* Δ*sigF*	1.17	3.42	2.18	0.12	1.56	8.46	0.232
Δ*ldhL* Δ*sigF* Δ*pdhA*	1.03	-1.33	6.43	nd[2]	0.15	7.60	0.236

Cells were grown for 22.5 h (wild-type, single and double mutant) or 28 h (triple mutant) in 1 L TVMY medium containing 30 g/L glucose in a 2 L reactor at 55°C and pH 6.5, without any gas additions.

[1] Abbreviations: Lac: lactate, Ace: acetate, Pyr: pyruvate, Mal: malate, Suc: succinate.

[2] nd: not determined: in the case of strain Δ*ldhL* Δ*sigF* Δ*pdhA*, malate could not be separated precisely from pyruvate due to high pyruvate concentrations.
